# Influence of cytochrome P450 polymorphisms on the antiplatelet effects of prasugrel in patients with non-cardioembolic stroke previously treated with clopidogrel

**DOI:** 10.1007/s11239-018-1714-2

**Published:** 2018-08-03

**Authors:** Takanari Kitazono, Yasuo Ikeda, Masakatsu Nishikawa, Satoshi Yoshiba, Kenji Abe, Akira Ogawa

**Affiliations:** 1 Department of Medicine and Clinical Science, Graduate School of Medical Sciences, Kyushu University, 3-1-1 Maidashi, Higashi-ku, Fukuoka, 812-8582 Japan; 2 Faculty of Science and Engineering, Waseda University, Tokyo, Japan; 3 Clinical Research Support Center, Mie University Hospital, Tsu, Mie Japan; 4 Daiichi Sankyo Co., Ltd, Tokyo, Japan; 5 Iwate Medical University, Morioka, Iwate Japan

**Keywords:** Prasugrel, Clopidogrel, CYP2C19, PRU, Stroke

## Abstract

This randomized double-blind crossover study aimed to investigate the influence of cytochrome P450 (CYP) 2C19 polymorphisms on the antiplatelet effects of prasugrel in patients with non-cardioembolic stroke treated with clopidogrel. Patients received clopidogrel 75 mg/day for > 4 weeks. Subsequently, patients received prasugrel 3.75 mg/day (group A; *n* = 64) or 2.5 mg/day (group B; *n* = 65) for 4 weeks followed by a 4 week switched-dose regimen. To assess the influence of CYP2C19 polymorphisms, patients were classified as extensive metabolizers (EMs), intermediate metabolizers (IMs), and poor metabolizers (PMs). The primary endpoint was P2Y_12_ reaction units (PRU) at the end of each 4 week treatment. A significant reduction in PRU was noted after treatment with prasugrel 3.75 mg/day compared with the pre-dose value (after treatment with clopidogrel) (p < 0.0001). By CYP2C19 phenotypes, a significant reduction in PRU was noted in IMs and PMs after treatment with prasugrel 3.75 mg/day and in PMs after treatment with prasugrel 2.5 mg/day, as compared with the pre-dose value (p < 0.0001). The plasma concentration of the active metabolite of clopidogrel was relatively low in PMs compared to EMs and IMs; prasugrel was similar across all CYP2C19 phenotypes. No major or clinically significant hemorrhagic adverse events occurred. By CYP2C19 phenotype, the antiplatelet effects of prasugrel were greater with 3.75 mg/day in IMs and PMs, and with 2.5 mg/day in PMs compared with clopidogrel 75 mg/day, without safety concerns. CYP2C19 polymorphisms did not affect the plasma concentration of the active metabolite of prasugrel or its antiplatelet effects. (JapicCTI-101044).

**Electronic supplementary material:**

The online version of this article (10.1007/s11239-018-1714-2) contains supplementary material, which is available to authorized users.

## Highlights

- The influence of cytochrome P450 (CYP) polymorphisms on the antiplatelet effects of prasugrel was investigated
- The active metabolites of prasugrel and clopidogrel have a similar degree of antiplatelet activity
- Compared to clopidogrel, prasugrel treatment provided equivalent antiplatelet effects at 2.5 mg/day and greater effects at 3.75 mg/day, without safety concerns
- CYP2C19 polymorphisms did not affect the antiplatelet effects of prasugrel
- Prasugrel may improve treatment outcomes in poor- or non-responders to clopidogrel, but further studies are necessary for confirmation

## Introduction

For secondary prevention of non-cardioembolic stroke, guidelines for the management of stroke recommend antiplatelet therapy, including clopidogrel and aspirin, which are currently used worldwide [1–3]. Although such antiplatelet therapy reduces the incidence of recurrent vascular events in patients with stroke or transient ischemic attack [4, 5], there are poor responders to aspirin and clopidogrel who remain at high risk for atherothrombotic events under antiplatelet therapy [6–10].

Clopidogrel is a prodrug requiring transformation to an active metabolite through hepatic metabolism by cytochrome P450 (CYP) enzymes (particularly CYP2C19). Therefore, CYP2C19 polymorphisms can cause reduced antiplatelet effects resulting from the decreased metabolic activation of clopidogrel, and consequently exposure to its active metabolite is decreased [11–20]. The presence of one or more reduced-function CYP2C19 alleles in patients receiving clopidogrel is shown to be associated with an increased risk of major cardiovascular adverse events (AEs) [6, 21]. Therefore, to improve treatment outcomes in poor- or non-responders to clopidogrel, there is a need for an antiplatelet agent that is not affected by CYP polymorphisms.

Prasugrel is a thienopyridine agent that, like clopidogrel, exerts its antiplatelet effects via selective inhibition of P2Y_12_ receptors. Prasugrel is also a prodrug and needs to undergo metabolism to its active form. Prasugrel is rapidly hydrolyzed in the intestine to a thiolactone, which is then converted to the active metabolite in a single step, primarily by CYP3A4 and CYP2B6, and to a lesser extent by CYP2C9 and CYP2C19 [20]. Thus, prasugrel has more consistent effects than clopidogrel, although the active metabolites of prasugrel and clopidogrel have a similar degree of antiplatelet activity [22].

The large-scale TRITON-TIMI 38 study showed that prasugrel (60 mg loading dose followed by a 10 mg maintenance dose) significantly reduced the rates of major cardiac events compared with clopidogrel in patients with acute coronary syndrome scheduled for percutaneous coronary intervention [23]. However, as the study showed higher incidences of major adverse cardiovascular events and bleeding events in patients with a history of stroke or transient ischemic attacks compared with clopidogrel, prasugrel is contraindicated in this group of patients in Western countries. In Japan, the approved doses of prasugrel (loading dose 20 mg, maintenance dose 3.75 mg) are lower than those used in Western countries.

Prasugrel (2.5, 5, and 7.5 mg once daily) is shown to be well tolerated and efficacious for inhibition of platelet aggregation in Japanese patients with non-cardioembolic stroke, and it has more potent antiplatelet effects than clopidogrel 75 mg once daily (unpublished observations, submitted). However, because of the small number of participants in that study (< 20 in each treatment group), it was not possible to compare the influence of CYP polymorphisms on the antiplatelet effects of prasugrel and clopidogrel in this subgroup of patients. Therefore, in the present larger-scale study, we aimed to investigate the influence of CYP2C19 polymorphisms on the antiplatelet effects of clopidogrel and prasugrel in patients with non-cardioembolic stroke who were previously treated with clopidogrel.

## Patients and methods

### Study design

This was a multicenter randomized double-blind two-way crossover study (Supplemental Figure). The study consisted of an observation period (pretreatment period, 4 weeks) followed by two consecutive treatment periods (periods 1 and 2; total 8 weeks). During the observation period (i.e. before the start of treatment with prasugrel), patients received clopidogrel 75 mg/day for > 4 weeks. Patients then received prasugrel 3.75 mg/day (group A) or 2.5 mg/day (group B) for 4 weeks (treatment period 1). Subsequently, the patients in group A were switched to prasugrel 2.5 mg/day and those in group B to prasugrel 3.75 mg/day for a further 4 weeks (treatment period 2). Prasugrel was taken orally once daily after breakfast. Each treatment period was defined as 4 weeks to enable the pharmacodynamic assessment to be done twice during each treatment period. For pharmacokinetic assessment, the plasma concentrations of the active metabolites of the drugs were measured once in each period (i.e. observation period and treatment periods 1 and 2).

The study was carried out at 14 hospitals in Japan between February 2010 and August 2010, in accordance with the Declaration of Helsinki, the Pharmaceutical Affairs Law, and Good Clinical Practice. The study protocol was approved by the institutional review board at each hospital, and written informed consent was obtained from each participant.

### Study population

The inclusion criteria were chronic stroke (excluding cardioembolic stroke and asymptomatic stroke), with the most recent ischemic stroke having occurred at least 4 weeks earlier; age, 20–74 years at the time informed consent was obtained; body weight, > 50 kg; and previous treatment with clopidogrel (75 mg/day) for ≥ 2 weeks for secondary prevention of ischemic stroke (patients who had received concomitant treatment with aspirin were excluded).

### Pharmacodynamic assessment

The primary endpoint was P2Y_12_ reaction units (PRU). PRU is a measure of platelet activity determined by using the VerifyNow^®^ assay (Werfen and Instrumentation Lab, Bedford, MA, USA) [24]. To determine PRU, blood samples were taken during the observation period, at 2 weeks before the start of prasugrel treatment; at baseline (week 0); and at 2, 4, 6, and 8 weeks after the start of prasugrel treatment. PRU testing was performed by each study site.

### Pharmacokinetic assessment

To measure the plasma concentrations of the active metabolites of prasugrel and clopidogrel (R-138727 and R-130964, respectively), blood samples were collected at 0.5, 1, 2, and 4 h after administration of each drug. At each sampling point, 5 mL of venous blood was collected into a vacuum blood sampling tube containing EDTA sodium. Immediately after blood sampling, 25 µL of 0.5 mol/L 3′-methoxyphenacyl bromide/acetonitrile solution was added, and it was mixed by inverting, and chilled on ice. Plasma obtained from centrifugation (4 °C, 3000 rpm, 10 min) was transferred into storage containers, and stored frozen (below − 20 °C). The plasma concentration of active metabolite of prasugrel and clopidogrel was measured using a liquid chromatography–tandem mass spectrometry (LC–MS/MS) method.

### Pharmacogenetic analysis

Genomic DNA from blood samples taken during the observation period and at 4 weeks before the start of prasugrel treatment were investigated for the presence of genetic polymorphisms of CYP2C19. Patients were classified into the following three groups according to CYP2C19 metabolizer phenotype: extensive metabolizers (EMs), with no mutant alleles; intermediate metabolizers (IMs), with one mutant allele; and poor metabolizers (PMs), with two or more mutant alleles.

To determine the influence of CYP polymorphisms on the antiplatelet effects of prasugrel and clopidogrel, PRU values and plasma concentrations of active metabolites were compared between the CYP metabolizer phenotype groups.

### Safety assessment

Data for reported incidences of AEs, including hemorrhagic and thrombotic events, were collected for the assessment of safety.

### Statistical analysis

The planned number of patients was 100 (group A, *n* = 50; group B, *n* = 50). To investigate the influence of CYP2C19 metabolizer phenotypes on the antiplatelet effects of clopidogrel and prasugrel, each group included at least 10 patients who were CYP2C19 PMs.

The determination of sample size was done as follows. Based on the results of clinical studies conducted in Japan, the difference in platelet reactivity index between CYP2C19 IMs and PMs after administration of prasugrel 2.5 and 3.75 mg/day was estimated to be 10%, and the intra-individual SD was estimated to be 15%. Therefore, a sample size of 50 patients (25 in each group) was calculated as the minimum number required to yield a power of 90% with a two-sided significance level of 5%. When the difference in platelet reactivity index between the screening period and the administration of prasugrel at either dose was ≥7.1%, the power was ≥90%, with a two-sided significance level of 5%. In consideration of the percentage of CYP2C19 EMs (50%), a target sample size of 100 (50 in each group) was chosen.

The full analysis set excluded patients for whom there was a major violation of the clinical study protocol (e.g. non-compliance with informed consent), patients who did not receive the study drug, and patients for whom no data were obtained after the study treatment. The per protocol analysis set comprised patients included in the full analysis set, who additionally were assessed at each visit as having drug adherence ≥70%, received the study treatment for five consecutive days (including the final day, in both treatment periods), completed treatment periods 1 and 2, and had no major violations of the clinical study protocol (e.g. violations of inclusion or exclusion criteria).

AEs were recorded according to the Medical Dictionary for Regulatory Activities version 13.0, and the number of patients with AEs and the numbers of AEs were tabulated by event. Statistical tests were carried out by using the SAS System Release 8.2.

For the pharmacodynamic assessment, the summary statistics of PRU measurements and changes from pre-dose were calculated by dose for each period (i.e. observation period and treatment periods 1 and 2). Longitudinal plots of mean values (with standard deviations) were prepared to show the PRU. For the changes in PRU from pre-dose, 95% confidence intervals (CIs) were also calculated. Similar analyses were done by CYP2C19 phenotype. For pharmacokinetic assessment, box plots of area under the curve (AUC) 0.5–4 h for the active metabolites (R-138727 and R-13096) were prepared for each drug, by CYP2C19 phenotype.

## Results

### Subject disposition and baseline characteristics

**Fig. 1:**
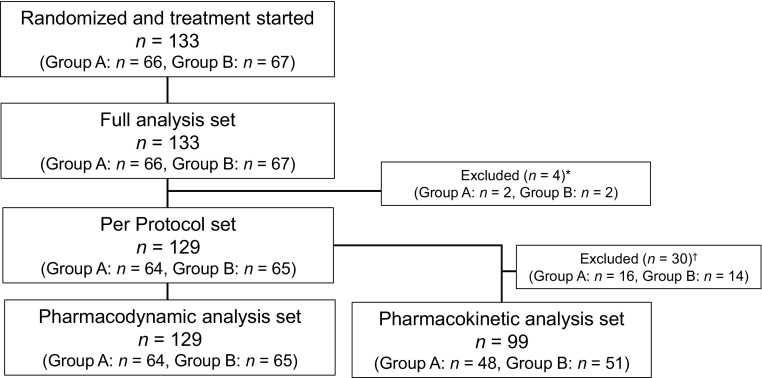
Patient disposition. *Subjects who did not receive study treatment for five consecutive days including the final day of study treatment in either period 1 or period 2. ^†^The reason for exclusion from the pharmacokinetic analysis set was that subjects in the PPS who were excluded did not have available pharmacokinetic data

Figure 1 shows the subject disposition. The per protocol analysis included data from 129 patients who were administered the study drug in accordance with protocol requirements and completed both treatment periods 1 and 2.

The baseline characteristics were similar between groups A and B (Table 1). In the total population, the mean age was 63.7 ± 7.7 years and the mean body weight was 66.4 ± 9.7 kg. The ratios of patients with large artery atherosclerosis and small artery occlusion were 29.5 and 69.8%, respectively. Regarding complications, 80.6, 62.8 and 24.0% of patients had hypertension, hyperlipidemia and diabetes mellitus, respectively. Of the total 129 patients in the per protocol analysis set, 36 were CYP2C19 PMs; the proportions of CYP2C19 PMs were similar between group A and group B (28.1 and 27.7%, respectively). Patients with other CYP metabolizer phenotypes were also similarly distributed between the two groups.

**Table 1:** Baseline characteristics (per protocol analysis set)

	Group A (*n* = 64)	Group B (*n* = 65)	p-value*
Sex			0.3491
Male	49 (76.6)	45 (69.2)	
Age (years) (mean ± SD)	62.8 ± 8.1	64.5 ± 7.2	0.2170
Weight (kg) (mean ± SD)	66.3 ± 9.7	66.5 ± 9.8	0.8918
Body mass index (kg/m^2^) (mean ± SD)	25.0 ± 3.0	25.4 ± 3.4	0.5023
Smoking habit			0.7033
Present	13 (20.3)	15 (23.1)	
Subtype of last stroke			
Atherothrombotic stroke	18 (28.1)	20 (30.8)	0.5650
Lacunar stroke	46 (71.9)	44 (67.7)	
Unknown	0 (0.0)	1 (1.5)	
Severity of disability (modified Rankin scale)			
Grade 0	31 (48.4)	24 (36.9)	0.4202
Grade 1	25 (39.1)	32 (49.2)	
Grade 2	7 (10.9)	6 (9.2)	
Grade 3	1 (1.6)	3 (4.6)	
Complications			
Hypertension	51 (79.7)	53 (81.5)	0.7903
Hyperlipidemia	38 (59.4)	43 (66.2)	0.4258
Diabetes mellitus	13 (20.3)	18 (27.7)	0.3267
CYP2C19 phenotype			0.9911
EMs	19 (29.7)	20 (30.8)	
IMs	27 (42.2)	27 (41.5)	
PMs	18 (28.1)	18 (27.7)	
IMs + PMs	45 (70.3)	45 (69.2)	

Values are expressed as the number (%), unless otherwise indicated

*EMs* extensive metabolizers; *IMs* intermediate metabolizers; *PMs* poor metabolizers

*Student’s *t* test was used for continuous variables, and Chi square test was used for categorical variables

No significant differences in the patient characteristics were noted between groups A and B in the full analysis set, the pharmacodynamic and pharmacokinetic assessment populations, and the safety analysis population.

### Primary endpoint

**Fig. 2:**
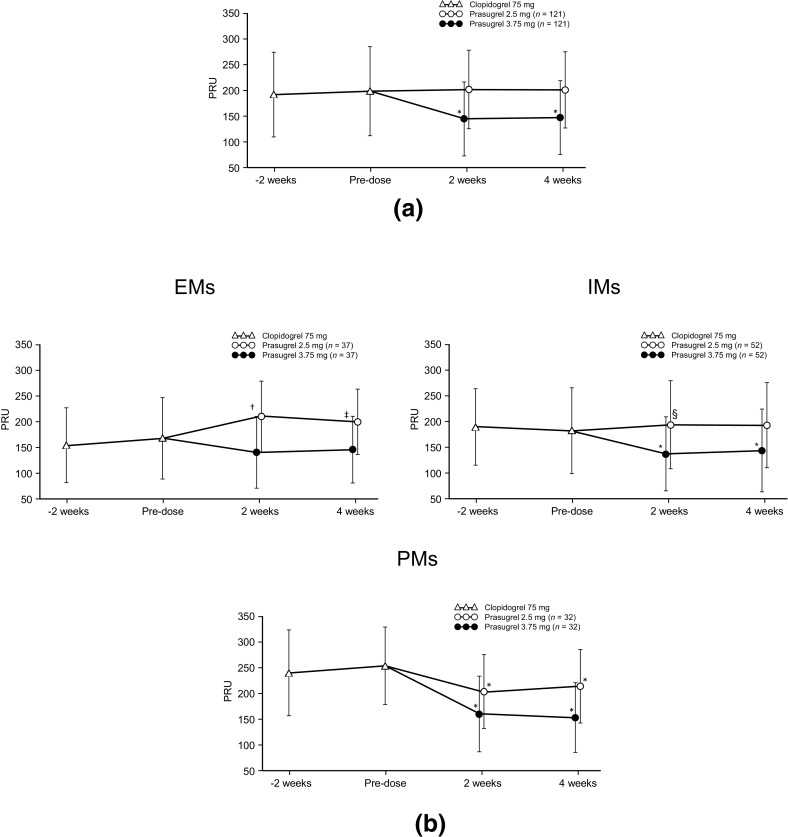
Longitudinal plots of mean values (with standard deviations) showing PRU in the total population (**a**) and by CYP2C19 phenotype (**b** EMs, IMs and PMs). The PRU level during administration of clopidogrel 75 mg (pre-dose of prasugrel) indicates the trough level. *p < 0.0001; ^†^p = 0.0001; ^‡^p = 0.0071; ^§^p = 0.0451 versus pre-dose value. *EMs* extensive metabolizers; *IMs* intermediate metabolizers; *PMs* poor metabolizers

The PRU (arithmetic mean ± SD) during the observation period, when patients received clopidogrel treatment, was 198.2 ± 86.7. After 4 week treatment with prasugrel 2.5 and 3.75 mg/day (combining the results of groups A and B), PRU was 200.9 ± 74.0 and 147.1 ± 71.6, respectively; a significant reduction in PRU was noted after treatment with prasugrel 3.75 mg/day, as compared with the pre-dose value (after treatment with clopidogrel) (p < 0.0001), whereas no significant difference was observed after treatment with prasugrel 2.5 mg/day (Fig. 2a).

Figure 2b shows the results of the subsequent analysis of PRU by CYP2C19 phenotypes. After treatment with prasugrel 3.75 mg/day, a significant reduction in PRU was noted in IMs and PMs compared with the pre-dose value (after treatment with clopidogrel) (at 2 and 4 weeks, p < 0.0001), whereas no significant difference was found in EMs. After treatment with prasugrel (2.5 mg/day), a significant increase in PRU was found in EMs (at 2 weeks, p = 0.0001; at 4 weeks, p = 0.0071) and in IMs (at 2 weeks, p = 0.0451), and a significant decrease was found in PMs (at 2 and 4 weeks, p < 0.0001). Additional subgroup analyses, with patients stratified by factors such as age, body weight, and sex, showed no particular effects on the results for PRU (data not shown).

### Pharmacokinetic outcomes

**Fig. 3:**
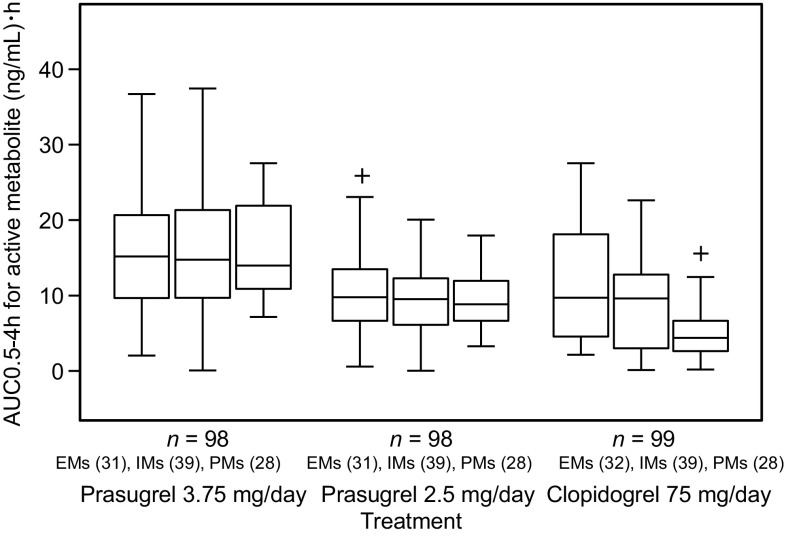
Area under the curve (AUC) 0.5–4 h for plasma concentration of the active metabolite of prasugrel in patients who received prasugrel 3.75 and 2.5 mg/day, and the active metabolite of clopidogrel in patients who received clopidogrel 75 mg/day. ^+^Value between 1.5 and 3 times the interquartile range away from the box. *EMs* extensive metabolizers; *IMs* intermediate metabolizers; *PMs* poor metabolizers

Figure 3 shows the AUC of the active metabolites of clopidogrel and prasugrel by CYP2C19 phenotype. While the plasma concentration of the active metabolite of prasugrel was similar across EMs, IMs, and PMs, that of the active metabolite of clopidogrel was lower in PMs than in EMs and IMs.

### Safety

Hemorrhagic AEs were reported in 6.8% (9/133) of patients: 3.0% (4/132) of patients during treatment with prasugrel 2.5 mg/day, and 3.8% (5/133) of patients during treatment with prasugrel 3.75 mg/day. Most of these AEs were mild, and none necessitated permanent discontinuation of the study drug.

No significant differences were found between the two treatment periods in terms of the incidence or onset of AEs (data not shown).

## Discussion

In Japanese patients with non-cardioembolic stroke, 4 weeks of treatment with prasugrel 3.75 mg/day resulted in a significant reduction in PRU, compared with clopidogrel 75 mg/day, whereas prasugrel 2.5 mg/day had similar effects to clopidogrel. The antiplatelet effects of prasugrel were unaffected by previous treatment with clopidogrel or the order in which patients received each dose in this crossover study.

The analysis of PRU by CYP2C19 phenotypes showed that, compared with the pre-dose value, a significant reduction in PRU was noted in IMs and PMs after treatment with prasugrel 3.75 mg/day, and in PMs after treatment with prasugrel 2.5 mg/day. PRU in patients with EM versus IM/PM was almost the same with both prasugrel doses. This indicates that the antiplatelet effects of prasugrel were unaffected by CYP2C19 polymorphisms, whereas the effects of clopidogrel decreased with an increasing number of mutant alleles. The finding is consistent with a previous report, which showed that CYP2C19 polymorphisms are associated with a poor response to clopidogrel, but not to prasugrel [20]. Because the active metabolites of clopidogrel and prasugrel inhibit P2Y_12_ ADP receptors to a similar degree, the differences in the antiplatelet effects of each of these drugs may be attributable to the differences in plasma concentrations of their respective active metabolites [22]. In the present study, as shown by AUC, there was no difference in the plasma concentration of the active metabolite of prasugrel (R-138727) according to CYP2C19 phenotypes, whereas that of the active metabolite of clopidogrel (R-130964) was lower in PMs than in EMs and IMs (Fig. 3). The findings suggest that the reduced antiplatelet effects of clopidogrel may be the result of decreased exposure of platelets to its active metabolite [22].

No particular safety concerns were noted with the use of prasugrel 3.75 mg/day; incidences of AEs, for which a causal relationship with prasugrel could not be denied, were similar between treatment periods during which patients received 2.5 or 3.75 mg/day. No serious AEs related to the study drug were reported. Regarding thrombotic events, angina pectoris was reported in 1 patient during the prasugrel 2.5 mg/day treatment period. However, this patient’s chest symptoms, including respiratory discomfort, were considered to have been present since before participation in the study. Therefore, it was difficult to evaluate the relationship between the effects of prasugrel and the incidence of thrombotic events in the present study. The incidence of hemorrhagic AEs was similar regardless of the prasugrel dose, and no major or clinically significant hemorrhagic events were reported.

## Conclusions

In patients with non-cardioembolic stroke, who switched from treatment with clopidogrel 75 mg/day, 4 week prasugrel treatment provided equivalent antiplatelet effects at 2.5 mg/day and greater effects at 3.75 mg/day, without any safety concerns. By CYP2C19 phenotypes, the antiplatelet effects of prasugrel were greater at 3.75 mg/day in IMs and PMs, and at 2.5 mg/day in PMs compared with clopidogrel 75 mg/day. CYP2C19 polymorphisms did not affect the plasma concentration of the active metabolite of prasugrel or its antiplatelet effects, compared to clopidogrel.

## Electronic supplementary material

Below is the link to the electronic supplementary material.

Supplementary material 1 (PPTX 64 KB)
