# Supplementary material for: Influence of cytochrome P450 polymorphisms on the antiplatelet effects of prasugrel in patients with non-cardioembolic stroke previously treated with clopidogrel
Source: J Thromb Thrombolysis. 2018 Aug 3;46(4):488–95. doi: 10.1007/s11239-018-1714-2 (PMC6182384; doi:10.1007/s11239-018-1714-2)
Supplement: Supplementary file 1 — Supplementary material 1 (PPTX 64 KB) [file 11239_2018_1714_MOESM1_ESM.pptx]

## Slide 1
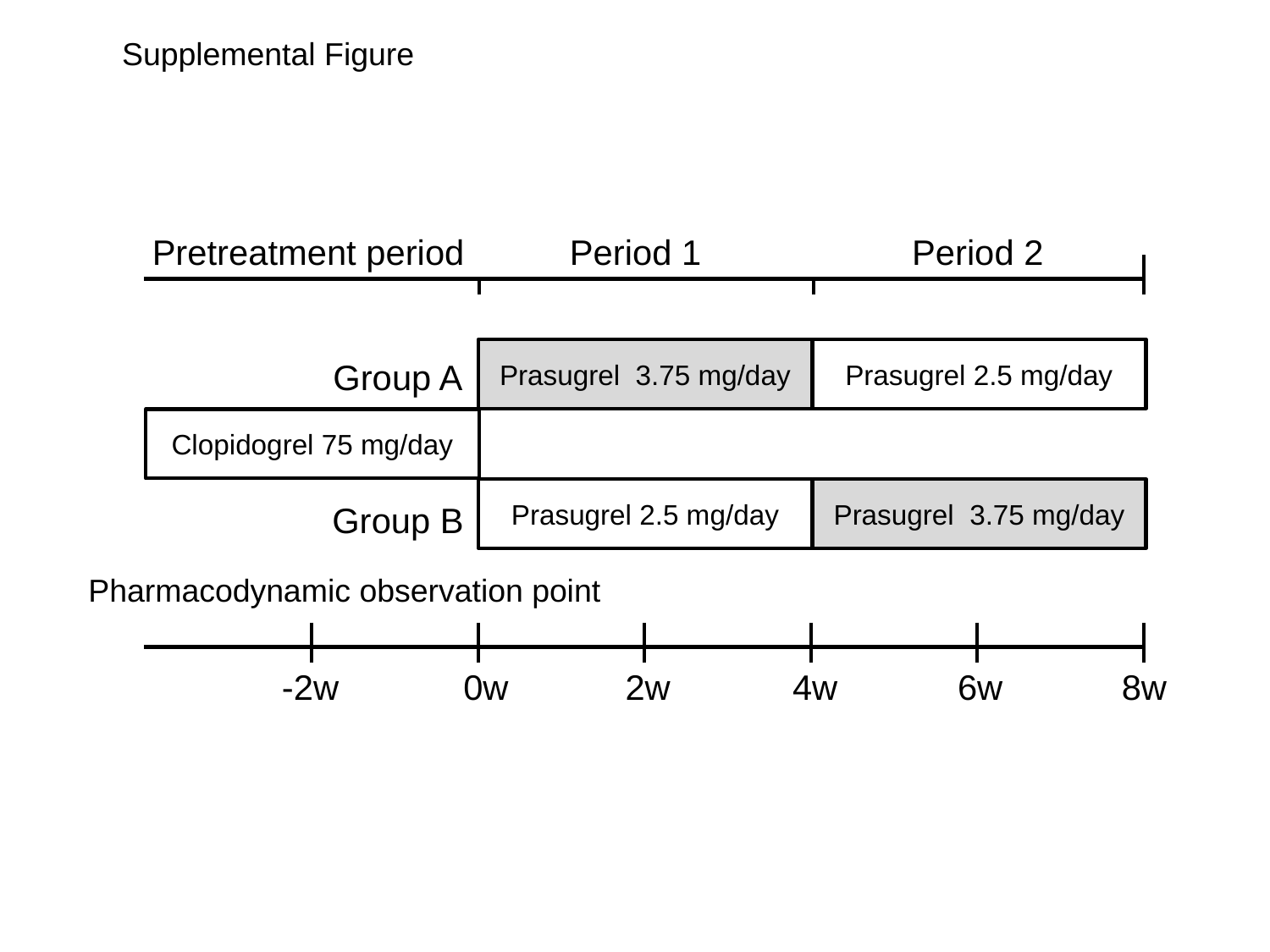

Supplemental Figure
Pretreatment period
Period 1
Period 2
Prasugrel 3.75 mg/day
Prasugrel 2.5 mg/day
Group A
Clopidogrel 75 mg/day
Prasugrel 2.5 mg/day
Prasugrel 3.75 mg/day
Group B
Pharmacodynamic observation point
-2w
0w
2w
4w
6w
8w
